# Supplementary material for: Dynamics of Urine Metabolomics and Tubular Inflammatory Cytokines in Type 1 Diabetes Across Disease Durations
Source: Metabolites. 2025 Nov 10;15(11):734. doi: 10.3390/metabo15110734 (PMC12654735; doi:10.3390/metabo15110734)
Supplement: Supplementary file 1 [file metabolites-15-00734-s001.zip › metabolites-3933942-supplementary.pdf]

Table S1. Results of Shapiro–Wilk normality tests for selected study variables.

Variables	DF	Statistic	p-value	Decision at level (5%)
MCP-1/Cr (µg/g)	247	0.64	0	Reject normality
KIM-1/Cr (µg/g)	247	0.21	0	Reject normality
NGAL/Cr (µg/g)	247	0.58	0	Reject normality
DM duration (years)	247	0.98	2.64E-04	Reject normality
UACR (mg/g)	247	0.22	0	Reject normality
Age (years)	247	0.99	0.31168	Can't reject normality
Height (cm)	247	0.90	9.29E-12	Reject normality
Weight (kg)	246	0.99	0.03203	Reject normality
BMI (kg/m^2)	247	0.98	5.68E-04	Reject normality
SBP (mmHg)	247	0.99	0.09579	Can't reject normality
DBP (mmHg)	247	0.99	0.01797	Reject normality
Cholesterol (mg/dL)	247	0.96	3.98E-06	Reject normality
LDL (mg/dL)	247	0.94	1.77E-08	Reject normality
HDL (mg/dL)	247	0.98	0.00493	Reject normality
Triglyceride (mg/dL)	247	0.59	0	Reject normality
BUN (mg/dL)	247	0.95	1.88E-07	Reject normality
Cr (mg/dL)	247	0.97	3.99E-05	Reject normality
eGFR (ml/min/1.73m^2)	247	0.93	1.90E-09	Reject normality
Uric acid (mg/dL)	247	0.97	7.15E-05	Reject normality
FBS (mg/dL)	247	0.94	6.43E-09	Reject normality
HbA1c (%)	247	0.90	1.10E-11	Reject normality
Homocysteine (µmol/L)	247	0.88	5.83E-13	Reject normality
hs CRP (mg/dL)	247	0.41	0	Reject normality
WBC (1000/µL)	247	0.89	1.75E-12	Reject normality
Hb (g/dL)	247	1.00	0.65459	Can't reject normality
Hct (%)	247	0.99	0.5222	Can't reject normality
Platelet (1000/µL)	247	0.33	0	Reject normality

A p-value < 0.05 indicates significant deviation from normality. These results justify the use of non-parametric statistical tests in the main analyses.

Table S2. Pearson correlation coefficients among renal biomarkers, markers of glucose metabolism, and metabolic and hematological parameters in the T1D-S, T1D-M, and T1D-L groups.

	Age at onset	UACR	eGFR	BMI	SBP	DBP	TC	LDL-C	HDL-C	TG	Uric acid	FBS	HbA1c	Homocysteine	hsCRP	WBC	Hb	Platelet
T1D duration	**0.73***	0.08	0.06	**0.43***	**0.37***	**0.32***	0.12	**0.14***	0.00	0.04	**0.17***	0.07	0.06	**0.23***	0.10	0.01	**0.18***	0.02
Age at onset	1.00	0.07	0.11	**0.56***	**0.44***	**0.45***	0.08	0.08	-0.01	0.07	**0.24***	-0.04	-0.01	**0.37***	0.11	-0.05	**0.19***	-0.02
UACR		1.00	0.03	-0.01	-0.08	0.00	0.06	0.06	-0.03	0.08	-0.01	0.02	**0.36***	0.06	0.08	0.07	-0.09	0.02
eGFR			1.00	-0.03	**-0.18***	-0.03	0.12	0.08	0.06	0.09	**-0.18***	-0.10	**0.19***	-0.09	0.02	0.11	**-0.22***	0.12
BMI				1.00	**0.51***	**0.44***	**0.17***	**0.23***	-0.07	0.11	**0.29***	**0.16***	0.05	**0.20***	**0.16***	**0.16***	**0.19***	0.04
SBP					1.00	**0.74***	0.00	0.01	-0.02	0.03	**0.33***	0.06	0.00	**0.25***	**0.15***	0.12	**0.33***	-0.01
DBP						1.00	0.10	0.10	-0.01	0.05	**0.25***	**0.13***	0.12	**0.20***	0.08	**0.24***	**0.26***	0.03
TC							1.00	**0.88***	**0.38***	**0.41***	-0.02	**0.20***	**0.37***	**-0.15***	**0.15***	**0.13***	-0.08	0.06
LDL-C								1.00	-0.01	**0.35***	0.02	**0.20***	**0.38***	-0.08	**0.17***	**0.19***	-0.02	0.04
HDL-C									1.00	**-0.22***	**-0.13***	0.07	0.00	**-0.16***	-0.03	-0.11	-0.06	0.05
TG										1.00	**0.13***	0.12	**0.32***	-0.04	**0.13***	0.08	-0.11	0.05
Uric acid											1.00	0.09	0.08	**0.27***	0.08	0.00	**0.41***	-0.02
FBS												1.00	**0.33***	-0.02	0.04	0.05	0.00	0.00
HbA1c													1.00	-0.11	**0.17***	**0.27***	0.03	**0.20***
Homocysteine														1.00	0.01	-0.08	**0.36***	**-0.13***
hsCRP															1.00	0.09	0.06	-0.01
WBC																1.00	0.05	0.07
Hb																	1.00	-0.07
Platelet																		1.00

*Correlation is significant at the 0.05 level (2 tailed).

Table S3. Logistic regression analyses of urinary cytokines (uMCP-1/Cr, uKIM-1/Cr and uNGAL/Cr) and covariates associated with albuminuria.

Variable	Adjusted OR (95% CI)	P-value
uMCP-1/Cr (μg/g)	0.85 (0.09–7.80)	0.887
uKIM-1/Cr (μg/g)	0.74 (0.46–1.18)	0.209
uNGAL/Cr (μg/g)	0.99 (0.98–1.00)	0.078
HbA1c (%)	0.63–0.67 (0.51–0.83)	<0.001
Sex (female)	0.43–0.53 (0.17–1.40)	0.078–0.197
Age (years)	0.95 (0.85–1.06)	0.329–0.420
BMI (kg/m^2)	1.09–1.13 (0.94–1.33)	0.128–0.266
eGFR (ml/min/1.73m^2)	0.99 (0.98–1.00)	0.120–0.238
Diabetes duration (years)	0.95–0.98 (0.85–1.09)	0.423–0.711

Binary logistic regression was performed with albuminuria (UACR ≥30 mg/g vs. <30 mg/g) as the dependent variable. Independent variables included urinary MCP-1, uKIM-1, or uNGAL (entered separately), along with HbA1c, age, sex, BMI, eGFR, and diabetes duration as covariates. Odds ratios (OR) with 95% CI and p-values are shown. A p-value <0.05 was considered statistically significant.

Table S4. Pearson correlation coefficients among renal biomarkers, markers of glucose metabolism, and metabolic and hematological parameters in male and female subjects.

	T1D duration	Age at onset	UACR	eGFR	BMI	SBP	DBP	TC	LDL	HDL	TG	Uric acid	FBS	HbA1c	Homocysteine	hsCRP	WBC	Hb	Platelet
T1D duration	1.00	**0.73***	0.08	0.06	**0.43***	**0.37***	**0.32***	0.12	**0.14***	0.00	0.04	**0.17***	0.07	0.06	**0.23***	0.10	0.01	**0.18***	0.02
Age at onset		1.00	0.07	0.11	**0.56***	**0.44***	**0.45***	0.08	0.08	-0.01	0.07	**0.24***	-0.04	-0.01	**0.37***	0.11	-0.05	**0.19***	-0.02
UACR			1.00	0.03	-0.01	-0.08	0.00	0.06	0.06	-0.03	0.08	-0.01	0.02	**0.36***	0.06	0.08	0.07	-0.09	0.02
eGFR				1.00	-0.03	**-0.18***	-0.03	0.12	0.08	0.06	0.09	**-0.18***	-0.10	**0.19***	-0.09	0.02	0.11	**-0.22***	0.12
BMI					1.00	**0.51***	**0.44***	**0.17***	**0.23***	-0.07	0.11	**0.29***	**0.16***	0.05	**0.20***	**0.16***	**0.16***	**0.19***	0.04
SBP						1.00	**0.74***	0.00	0.01	-0.02	0.03	**0.33***	0.06	0.00	**0.25***	**0.15***	0.12	**0.33***	-0.01
DBP							1.00	0.10	0.10	-0.01	0.05	**0.25***	**0.13***	0.12	**0.20***	0.08	**0.24***	**0.26***	0.03
TC								1.00	**0.88***	**0.38***	**0.41***	-0.02	**0.20***	**0.37***	**-0.15***	**0.15***	**0.13***	-0.08	0.06
LDL									1.00	-0.01	**0.35***	0.02	**0.20***	**0.38***	-0.08	**0.17***	**0.19***	-0.02	0.04
HDL										1.00	**-0.22***	**-0.13***	0.07	0.00	**-0.16***	-0.03	-0.11	-0.06	0.05
TG											1.00	**0.13***	0.12	**0.32***	-0.04	**0.13***	0.08	-0.11	0.05
Uric acid												1.00	0.09	0.08	**0.27***	0.08	0.00	**0.41***	-0.02
FBS													1.00	**0.33***	-0.02	0.04	0.05	0.00	0.00
HbA1c														1.00	-0.11	**0.17***	**0.27***	0.03	**0.20***
Homocysteine															1.00	0.01	-0.08	**0.36***	**-0.13***
hsCRP																1.00	0.09	0.06	-0.01
WBC																	1.00	0.05	0.07
Hb																		1.00	-0.07
Platelet																			1.00

*Correlation is significant at the 0.05 level (2 tailed).
